# Enhancing Pectin Particles with Polymer Additives: Mitigating Rumen Degradation and Minimizing Yellowish Milk Color in Grazed Cows

**DOI:** 10.3390/polym16010106

**Published:** 2023-12-29

**Authors:** Francisco Vera-Vázquez, Jacinto Efrén Ramírez-Bribiesca, Rosy G. Cruz-Monterrosa, María M. Crosby-Galvan, José Ricardo Barcena-Gama, Diana Tamara Ramírez, Jorge L. Mejía-Méndez, Laura H. Vallejo-Hernández, Edgar R. López-Mena

**Affiliations:** 1 Programa de Ganadería, Colegio de Postgraduados, Km. 36.5, Montecillo, Texcoco 56230, Estado de México, Mexico; vera.francisco@colpos.mx (F.V.-V.); maria@colpos.mx (M.M.C.-G.); rbarcena@colpos.mx (J.R.B.-G.); 2 División de Ciencias Biológicas y de la Salud, Departamento de Ciencias de la Alimentación, Universidad Autónoma Metropolitana, Unidad Lerma, Av. Hidalgo Poniente 46, Col. La Estación, Lerma de Villada 52006, Estado de México, Mexico; 3 Department of Chemistry, UC Davis, 1 Shields Ave, Davis, CA 95616, USA; dtramirez@ucdavis.edu; 4 Laboratorio en Investigación Fitoquímica, Departamento de Ciencias Químico-Biológicas, Universidad de las Américas Puebla, Ex Hacienda Sta. Catarina Mártir S/N, Puebla 72810, San Andrés Cholula, Mexico; jorge.mejiamz@udlap.mx; 5 Departamento de Enseñanza, Investigación y Servicio en Zootecnia, Universidad Autónoma Chapingo, Km. 38.5 Carretera México—Texcoco, Chapingo, Texcoco 56230, Estado de México, Mexico; lvallejoh@chapingo.mx; 6 Escuela de Ingeniería y Ciencias, Campus Guadalajara, Tecnológico de Monterrey, Av. Gral. Ramón Corona No 2514, Zapopan 45121, Colonia Nuevo México, Mexico; edgarl@tec.mx

**Keywords:** pectin, protection, palm oil, calcium oxide, shellac, microparticles

## Abstract

The pigments consumed in grazing give the milk from dual-purpose cows raised in tropical conditions a yellowish color, affecting the quality and price of the milk. This study aimed to develop an economical method with supplementary pectin to antagonize the availability of carotenes by designing microparticles with shellac and palm oil as a viable alternative to protect pectin degradation against rumen microbes. Three preparations of microparticles based on citrus pectin were synthesized: unprotected (PnP), protected with palm oil (PwP), and protected with palm oil and shellac (PwPL) microparticles. Samples were roughly characterized by spectroscopy and electron microscopy techniques. The effect of PnP, PwP, and PwPL on blood metabolites and physicochemical characteristics of the milk of grazing lactating cows was evaluated through in vivo assays. The release of citrus pectin from microparticles was determined as uronic acids using solutions with distinct pH, whereas its degradation was studied using in situ tests. Results revealed that PnP, PwP, and PwPL are amorphous structures with sizes that range from 60 to 265 nm or 750 to 3570 µm and have surface charges that range from −11.5 to −50.2 mV. Samples exhibited characteristic peaks during FTIR analyses that corresponded to O-H, C=O, and COOCH_3_ groups and bands within the UV-vis region that indicated the absorption of pectin. The EDS analysis revealed the presence of carbon, oxygen, or calcium in samples. The release of uronic acids was higher at pH 2–3 with PwPL. The in situ degradability of PnP, PwP, and PwPL was 99, 28.4, and 17.7%, respectively. Moreover, PwPL decreased the blood concentration of glucose, cholesterol, and lactate. In contrast, 100 g of pectin per animal daily during the feed process reduced yellow coloring. In conclusion, designing particles protected with lipids and polymers as shellac is an economical method that resists degradation at pH levels greater than five.

## 1. Introduction

Microparticles are materials found within the 1–1000 mm range. They can be synthesized with natural (e.g., agarose and chitosan), synthetic (e.g., poly lactone and poly orthoester), or semisynthetic (e.g., cellulose acetate and hydroxymethyl cellulose) polymers, and inorganic materials such as silver and copper [1]. Given the processing parameters, microparticles can be synthesized by spray drying [2], in situ polymerization [3], biological techniques, precipitation, and slow carbonation [4]. In contrast to nanometric scale objects, these structures’ micrometric architecture facilitates their handling and controlled release of additives, antimicrobials, flavoring agents, pigments, and fertilizers [5,6]. The encapsulation efficiency, release, permeability, solubility, and desired performance of microcapsules can vary in accord with the components of shell materials and their properties [1]. It can be modified to design and apply innovative materials to solve human health threats.

The increase in the human population impacts on the demand for meat and milk, improving efficiency in ruminant production [7]. Currently, animal-derived products must comply with quality and safety standards. For instance, milk’s color influences farmers’ organoleptic and economic characteristics worldwide.

Color is a visual measure reflecting freshness, quality, and acceptance [8]. Specifically, the yellowish-white color in bovine milk is attributed to the size of the fat globules and the amount of β-carotenes, along with small quantities of riboflavin, lutein, zeaxanthin, and xanthophylls.

Grazing cows consume significant amounts of green forage rich in carotenes [9]. However, unlike sheep and goats, cows lack the intestinal 15-15-dioxygenase enzyme [10], preventing the conversion of carotenes into vitamin A. Physiologically, β-carotene is stored in the mammary gland and transferred to milk encapsulated within fat globules. During cheese production, the fat globules rupture, exposing β-carotene, which imparts the characteristic yellow color to the cheese [11]. The yellow color index is a biomarker to estimate carotenoid content, primarily β-carotene [12]. While most consumers prefer a slightly yellowish color in milk, an intense yellow hue is associated with poor quality or milk spoilage. Consequently, efforts are made to reduce the deposition of β-carotene in milk.

Pectin is a polymer with a linear structure formed by the galacturonic acid monomer units linked via α-(1→4)-glycosidic bond. Given its chemical structure, it can decrease the digestion of carotenoids by causing agglutination and increasing viscosity. This is primarily due to its high degree of methoxylation, which reduces the formation of micelles and restricts the movement of β-carotene in the villous intestinal epithelium. As a result, the conversion of β-carotene to vitamin A is diminished [13], and there is a negative correlation between the bioaccessibility of β-carotene and the pectin content [13,14]. Previous studies have indicated that pectin antagonistically affects carotenoid uptake and utilization in chickens and laboratory animals [13,15]. A study by Cruz et al. [16] observed a reduction in β-carotene digestion in bovines when pectin was infused at a rate of 92.5 g per day through a duodenal cannula. However, since rumen microorganisms readily degrade pectin, measures need to be taken to protect it so that it can reach the intestine and exert its antagonistic effect on β-carotene absorption [17,18]. The nutrients and polymers used in pectin encapsulation must be non-toxic, free from off-flavors for animals, readily available, and cost-effective [17,19,20]. Representative examples of this are palm oil and calcium oxide (CaO).

Palm oil is an edible oil obtained from the mesocarp of fruits of the palm tree *Elaeis guineensis*. Chemically, it is constituted by two major compounds: palm olein (65–70%) and stearin (30–35%) [21]. Due to the abundant presence of palm olein which is mainly conformed by carotenes and derivatives of vitamin E (tocotrienols and tocopherols), palm oil is characterized by its yellow to orange-red color. Palm oil is predominantly used in processed meals, snacks, and baking in the food industry due to its practicality, texture, and taste [22]. In animals, palm-oil-based formulations can enhance feed palatability, decrease the digesta passage rate, and up-regulate the high metabolizable energy of broiler chickens [23]. Comparably, it can be used as a superior supplemental additive to increase the milk fat percentage and yield in Holstein cows [24]. In contrast, CaO is a white-to-pale yellow commercial powder commonly used to manufacture Portland cement [25]. The scientific evidence regarding the use of CaO in livestock and agriculture is limited; however, it has been exploited to synthesize materials on the nanometric scale with the capacity to adsorb toxic chemical compounds and decrease the viability of bacteria strains [26].

Shellac is a natural resin that originated from the secretion of *Kerria lacca* constituted by shellolic acid, jalaric acid, and aleuritic acid [27]. The recent review by Thombare et al. [28] indicates shellac as a compound of monomers that integrate a moldable macromolecule under specific pressure and temperature conditions. It is edible, non-toxic, biodegradable, tasteless, soluble in alcohol, and water resistant. Because of these characteristics, shellac has been highlighted as a resin with traditional and modern applications, specifically developing innovative materials for the industry and environment.

Although no reference is made to its use in livestock, it can be an excellent polymer to cover substances or nutrients. The oral administration of additives for ruminants presents challenges in designing protection methods against rumen microorganisms while considering the release of pectin at a pH lower than five, as encountered in the intestinal tract [29]. The protected additives need to be effectively released in the small intestine, requiring the design of microcapsules, microspheres, nanoparticles, slow-release boluses, and other models with controlled release times and efficient dosing systems [18].

This study aimed to develop an economical method for protecting citrus pectin by utilizing combinations of protective materials such as palm oil, calcium oxide, and shellac. The unprotected citrus pectin microparticles were termed PnP, whereas citrus pectin microparticles protected with palm oil were designated PwP. In contrast, the citrus pectin microparticles protected with palm oil and shellac were named PwPL. The study was conducted in two phases: the first phase involved manufacturing and characterizing the protected citrus pectin particles. The second phase comprised an in vivo experiment conducted with grazing cows. The physical and chemical features of the produced microcapsules involved the use of spectroscopy and microscopy techniques. Spectroscopy methods included ultraviolet-visible (UV-vis) spectroscopy, Fourier-transformed infrared spectroscopy (FTIR), dynamic light scattering (DLS), and energy-dispersive X-ray spectroscopy (EDS), whereas microscopy analyses included scanning electron microscopy (SEM).

## 2. Materials and Methods

Four Holstein steers (300 ± 10.5 kg) were cared for following the Mexican Council on Animal Care guidelines to evaluate the capacity of the developed microparticles to mitigate rumen degradation and minimize yellowish milk color [30]. The ruminal and duodenal cannula procedures were performed through surgeries three months before the start of the experiment. The protocol for the maintenance and handling of the steers was approved by the Animal Care Administrative Advisory Committee of the Colegio de Postgraduados, student protocol, with an enrollment of 11,831,011.

### 2.1. Synthesis, Characterization, and In Vitro and In Vivo Analysis of PnP, PwP, and PwPL

The experimental design regarding the synthesis, characterization, and in vitro and in vivo evaluation of PnP, PwP, and PwPL was divided into five phases. The manufacturing process of these samples is represented in the Supplementary Figures (Figure S1).

In the first phase, microparticles of pectin were covered with palm oil and shellac, making it insoluble in the pH range of 5.8–6.5 and facilitating its degradation at pH < 4 to enhance its availability at the intestinal level as well as its binding with carotenes. The procedure performed to synthesize PnP microparticles with citrus pectin (69% esterification degree, Cargill^®^, Minneapolis, MN, USA) was published [31]. Briefly, 100 g of palm oil was weighed and slightly heated until it reached its melting point at 50 °C. Then, 100 g of pectin was added and homogenized. After that, CaO was added to hot African palm oil from *E. guineensis* in a 1:1 ratio. The mixture was further heated between 100 and 150 °C while applying pressure of 2 to 4 bars for 20 min utilizing a LAB-MED LMV40 autoclave (Cd. México, México). Simultaneously, 200 mL of distilled water was added to the mixture to allow CaO to form CaOH. This procedure resulted in the formation of PwP microparticles.

Furthermore, the dried PwP was infused with a shellac solution (Cedrosa S.A. Naucalpan, México) for 5 min. The shellac solution was prepared by mixing 200 g of shellac with 1 L of reagent-grade alcohol and stirring it for 20 min on a Thermo Scientific magnetic plate (Sp131635, China). This process enabled the production of PwPL microparticles. Samples were dried at room temperature for 24 h and stored in 10 kg plastic bags until their evaluation in the next phase.

The synthesized particles were characterized using UV-Vis spectroscopy, FTIR spectroscopy, DLS, EDS, and SEM. The UV-Vis spectroscopy analysis of PnP, PwP, and PwPL was performed using a Cary 60 UV-Vis spectrophotometer (Agilent Technologies, Santa Clara, CA, USA) in 1 cm quartz cuvettes from 200 to 800 nm. To evaluate samples by FTIR spectroscopy, an Agilent Technologies FTIR spectrometer (Santa Clara, CA, USA) was used, and measurements were determined within the 4000 to 400 cm^−1^ wavenumber region. The average particle size, size distribution, and ζ-potential of PnP, PwP, and PwPL were evaluated utilizing a Microtrac Nanotrac Wave II DLS (Montgomeryville, PA, USA). The EDS analysis of samples was executed using a Bruker XFlash 6|30 energy-dispersive X-ray detector (Berlin, Germany). For the SEM analysis, samples were placed in a brass sample holder and secured with double-adhesive aluminum and carbon conductive tape. The particles were coated with gold for 4 min using a JEOL JFC-1100 metal ionizer (Fine Coat^®^ ion Sputter, Mexico). Subsequently, the samples were observed and analyzed in a scanning electron microscope (JEOL-JSM 6390, Mexico) at 10 Kv.

In the second phase, in vitro measurements of the bioaccessibility rate between pectin and total carotenes were conducted to demonstrate that citrus pectin-based microparticles antagonize the absorption of carotenes. The in vitro bioaccessibility test was conducted using two groups: duodenal chyme carotenes (DC), which consisted of 50 mL of duodenal chyme, and duodenal chyme carotenes with pectin (DCP), which included 50 mL of duodenal chyme plus 20 mg of citrus pectin. Two reference controls were included: WC, which involved 50 mL of deionized water, and WCP, which included 50 mL of deionized water plus citrus pectin. The purpose of the reference controls was to eliminate the possibility of contamination with carotenes. All treatments were prepared in 200 mL Erlenmeyer flasks with 5 replicates per group.

Duodenal chyme was collected directly from two steers with T-cannulas fistulated in the proximal duodenum 2 h after feeding. The containers were immediately covered with aluminum foil and transported to the laboratory for allocation to the designated groups. The flasks were maintained at 37 °C with shaking at 90 rpm for 2 h [32]. Aluminum foil was also used to cover the flasks, preventing the denaturation of carotenoids caused by light exposure. Subsequently, all samples were centrifuged at 700 rpm in a dark room, and the supernatant was collected. The concentration of carotenes in the supernatant was measured at 454 nm using a Spectronic R 20 spectrophotometer (Milton Roy, Rochester, NY, USA), with ether used as a blank [33]. The evaluation of the *bioaccessibility* of PnP, PwP, and PwPl was calculated using Equation (1):

(1) $$Bioaccessibility\;\left(\%\right)=\frac{DCP\;content}{DC\;content}$$

The third phase consisted of an indirect method to evaluate the release rate of pectin at pH < 4.0 by quantifying uronic acids. Initially, the buffer solutions (pH 7 and 5) were prepared by mixing sodium bicarbonate (Fermont 12903-2.5), sodium phosphate heptahydrate (J.T. Baker 3828-01), potassium chloride (KCl) (J.T. Baker 3040-1), sodium chloride (NaCl) (Meyer 2365-500), and magnesium sulfate heptahydrate (J.T. Baker 2500) in 2 L of distilled water. McDougall’s artificial saliva was adjusted to pH 7.0–5.0 using acetic acid (J.T. Baker 9507-60). Furthermore, the pH 3.0 buffer was prepared by mixing 10.21 g of potassium acid phthalate (Mallinckrodt 5704) with 250 mL of water and 4.108 mL of hydrochloric acid (HCl) (J.T. Baker 9535-05) and making up the volume to 250 mL. To create the pH 2.0 buffer, 3.73 g of KCl was mixed with distilled water to reach a volume of 250 mL. Then, 100 mL of the KCl solution was mixed with 21.2 mL of the HCl solution, and the volume was adjusted to 400 mL for the degradability tests.

The release rate tests were divided into PnP, PwP, and PwPL treatments. Ten replicates of each treatment were prepared using 0.5 g of the sample in a 50 mL plastic tube. Subsequently, 25 mL of each buffer solution (pH: 2, 3, 5, 7) was added to the tubes, which were then placed in a glass jar in the Daisy Ankom digester (ANKOM Technology, D200, Rochester, NY, USA) at a temperature of 39 °C to simulate rumen conditions. The samples were agitated continuously. After 1 h, a 250 µL aliquot was taken from each sample and placed in an ice bath on a magnetic plate (Thermo Scientific, Sp131635, Fujian, China) for acid digestion. Each sample was treated with 1 mL of concentrated sulfuric acid (H_2_SO_4_) and stirred magnetically for 10 min. Then, 500 µL of distilled water was added to each sample and stirred for 10 min. This process was repeated with another addition of 500 µL of distilled water and stirring for 20 min. Finally, the samples were shaken and cooled with ice for 10 min before measuring the concentration of uronic acids.

To quantify the percentage of uronic acids, a 0.1 mL aliquot of each sample was placed in an ice bath, and 1 mL of a 0.5% sodium borate solution in concentrated H_2_SO_4_ was added. The resulting mixture was vortexed in a mixer (Labnet, So200, Edison, NJ, USA) and placed in boiling water (100 °C) for 5 min. The tubes were then placed on ice for 5 min, and 20 µL of 0.15% m-phenylphenol (Sigma Alldrich 45529-250 mg) in 0.5% sodium hydroxide was added. After vortexing, the tubes were left at room temperature (24–27 °C) for 15 min. After the resting period, the samples were shaken, and the absorbance at 520 nm was measured using a spectrophotometer (Bekman^®^ DU 65 UV VIS, Brea, CA, USA). The concentration was calculated using a calibration curve ranging from 0 to 40 µg of D-galacturonic acid (Sigma Aldrich 48280-5G-F, Saint Louis, MO, USA). The pectin released from PwP or PwPL was determined by calculating *the percentage of uronic* acids using Equation (2), where mg represents the value of the concentration of D-galacturonic acid, whereas DF constitutes the dissolution factor. The considered weight of the sample (mg sample) was 5000 mg.

(2) $$\%\;uronic\;acids=\left[\frac{\left(µg\right)(DF)}{µg\;\mathrm{sample}}\right]\times 100$$

The fourth phase included the determination of PnP, PwP, and PwPL degradation in bovines through in situ bags introduced into the rumen of cannulated bovines. Three Holstein bulls with a live weight of 500 ± 50 kg, equipped with fistulas and rumen cannulas, were used for the experiment. The evaluated treatments were as follows: (a) PnP, (b) PwP, and (c) PwPL at two degradability times, 24 and 48 h. For each treatment, 20 g of the sample was placed in a polyseda-type cloth bag (nylon^®^) measuring 10 × 20 cm with a pore size of 115 μm. These bags were then introduced into the rumen through the cannula [34]. Each treatment had 5 replicates, and 2 additional bags were used as blanks, resulting in 34 bags for each animal. After incubation in the rumen, the bags were removed and washed with running water. They were then dried in an oven (Riossa e-51, Cd. México, México) at 50 °C for 24 h. The degradability percentage of the protected citrus pectin was calculated according to Equation (3).

(3) $$In\;situ\;DM\;degradability=\left(\frac{initial\;DM\left(g\right)-residual\;DM\;(g)}{initial\;DM\;(g)}\right)\times 100$$

Finally, the fifth phase encompassed the in vivo test with grazing lactating cows, analyzing the obtained milk’s blood metabolites and physicochemical characteristics. The experimental design of this section was executed with the results obtained in the third and fourth phases, respectively. Even though the results do not exhibit differences between the two pectin encapsulation techniques, PwPL was selected for use in the animal experimental unit. In this regard, 20 cows, which were crossbreeds between zebu and Swiss, were used for the experiment. The cows had a mean weight of 400 ± 50 kg and were multiparous. They were grazing extensively and had access to colocho grass (*Digitaria swazilandensis*) and water ad libitum. The cows were randomly divided into two groups, with ten animals in each group. The treatments administered were as follows: microparticles, which did not include citrus pectin, and PwPL, which involved providing 100 g of protected citrus pectin per animal per day. The pectin particles were mixed with 250 g of commercial concentrate and offered to the cows after milking (7:00 a.m.). The experiment lasted 45 days.

For milk sampling, 100 mL of milk per cow was collected (twice a week) in 120 mL plastic bottles and stored at −4 °C until analysis. Before analysis, the samples were thawed at room temperature and then heated to 30 °C using a Felisa water bath (model FF372, 8 L capacity). Subsequently, the samples were transferred to a 250 mL flask and homogenized. A 15 mL portion of the sample was taken and placed in the sample holder of the Milkotester LTD^®^ Eco model. After 60 s, the percentage of fat, protein, total solids, lactose, and pH were recorded. The color of the milk was measured using a KONICA MI-NOLTA^®^ CR-410 precision colorimeter, which recorded values for CIEL* (luminosity), a* (red index), b* (yellow index), and saturation index C values (chroma, C*). The C* value is defined by the angle between a* and b* and is denoted as (a2 + b2) 1/2. The Brix degrees were quantified using a HANNA^®^ refractometer (model HI96801). Blood samples were collected on the final day of the experiment, 2 h after feeding, by puncturing the coccygeal vein using needles and 6 mL BD Vacutainer^®^ plastic tubes containing heparin sodium. Subsequently, the blood metabolites, including glucose, cholesterol, triglycerides, and lactate, were quantified using an Accutrend^®^ Plus portable meter.

### 2.2. Statistical Analysis

The data obtained from the in vitro release of pectin were analyzed using the SAS GLM procedure with a completely randomized design. The Tukey test (*p* < 0.05) compared the means between treatments. The in situ degradation data of the particles were analyzed using a completely randomized design with the SAS-ANOVA procedure. Tukey’s test (*p* < 0.05) compared means between treatments. The data about the physicochemical characteristics of milk and blood metabolites were analyzed using a completely randomized design with two treatments and ten repetitions. The comparison between treatments was performed using the t-Student test (*p* < 0.05).

## 3. Results

### 3.1. Characterization of PnP, PwP, and PwPL

**Figure 1:**
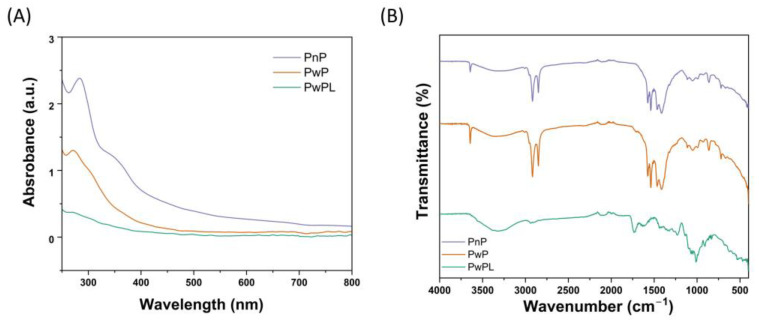
(**A**) “ UV—Vis ” spectroscopy and (**B**) FTIR spectroscopy analyses of PnP, PwP, and PwPL microparticles.

The characterization of microparticles through spectroscopy and microscopy techniques is necessary to determine their chemical composition, size, shape, and surface charge. UV-Vis spectroscopy is a routine analysis technique related to the amount of UV-Vis light absorbed by organic and inorganic samples. As depicted in Figure 1A, PnP microparticles exhibited a prominent absorption peak at 215 nm and a broad band at 350 nm. PwP microparticles presented a unique wide peak at 206 nm in the same range. Interestingly, these bands were not recorded during the UV-Vis analysis of PwPL microparticles, which can indicate that pectin is completely covered by shellac and palm oil.

As presented in Figure 1B, PnP, PwP, and PwPL microparticles exhibit a sharp peak at 3641 cm^−1^ and a wide band at 3326 cm^−1^, which can correspond to the stretching vibration of the O-H bond from pectin. In the same figure, it can be noted that the synthesized microparticles present two sharp peaks located at 2922 and 2853 cm^−1^, respectively. In the case of PwP and PwPL, the former can be related to the stretching of the triglyceride chains’ C–H bond from palm oil’s chemical structure. In comparison, the latter can be associated with the CH_2_ moieties of the fatty acid backbone of the same substance [35]. Compared to PwP and PwPL, PnP microparticles exhibit an evident small peak at 1725 cm^−1^, which can be related to the stretching of the C=O bond from the COOCH_3_ group of pectin [36]. The peaks distributed from 1571 to 827 cm^−1^ can represent the fingerprint region of PnP, PwP, and PwPL microparticles. Still, it has been documented that bands located at 1464 and 1408 cm^−1^ can be correlated to the bending vibration of the CH_2_ and CH_3_ moieties from shellac, respectively [37]. The peak observed at 720 cm^−1^ can be associated with the rocking of the CH_2_ group from shellac.

**Figure 2:**
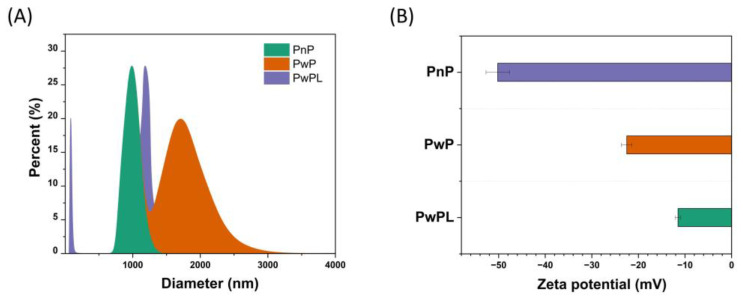
(**A**) DLS analysis and (**B**) ζ-potential (mV) of PnP, PwP, and PwPL microparticles.

As represented in Figure 2A, the DLS analysis revealed that the PnP sample comprehends a series of microparticles that range from 750 to 1375 nm. In addition, the PwP microparticles’ size goes from 1156 to 3570 nm, whereas the size distribution of PwPL microparticles goes from 265 to 60.80 nm. The average hydrodynamic size of PnP microparticles is 1017 nm (1.017 mm), whereas for PwP microparticles it is 1800 nm (1.8 mm). Similarly, the average hydrodynamic size of PwPL microparticles is 86.7 nm (0.0867 mm). In the same figure, it can be noted that the ζ-potential of the three samples was recorded. The calculated ζ-potentials of PnP, PwP, and PwPL microparticles were −50.2, −22.5, and −11.5 mV, respectively. The calculated PDI values of PnP, PwP, and PwPL microparticles were 0.009, 0.02, and 0.01, respectively.

**Figure 3:**
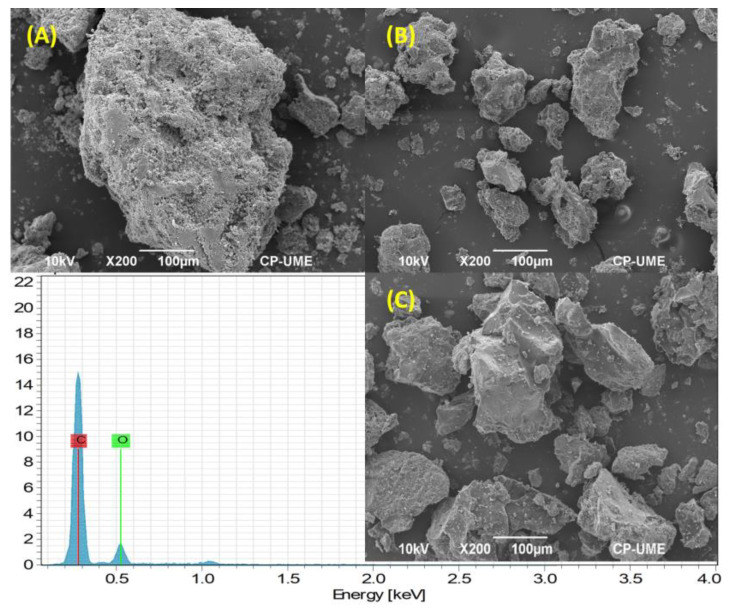
SEM analyses of (**A**) PnP, (**B**) PwP, and (**C**) PwPL microparticles.

According to Figure 3A, PnP microparticles showed a similar external topography, with round and oval shapes of porous consistency. The outer surface is continuous with slight cracks and fissures; some regions are wrinkled and hollow, facilitating the permeability and adhesion of other substances. The size was homogeneous, around 185 µm. In Figure 3B, it can be observed that PwP microparticles were more isolated and had irregular shapes, which can be attributed to the mixing process where ingredients compact and crystal-like shapes were formed. The sizes ranged from 70 to 200 µm. On the other hand, Figure 3C depicts that PwPL microparticles presented a more solid and less porous surface due to the covering and hardening of the layer caused by the polymer. Their structures are more compact and have square and hexagonal shapes.

Although there were several aggregates of smaller entities, the amorphous morphology of PwPL microparticles is defined as dispersions of solid particles, where the entrapped or encapsulated pectin is bound to a multi-particle matrix due to polymer binding. A single layer of shellac appreciably changed the appearance of the particle; the size of these microparticles ranged from 91 to 152 µm. The images show that the shellac coating appears slightly lighter in color, completely covering the pectin. As expected, PnP, PwP, and PwPL microparticles manifested the presence of carbon and oxygen, the main elements that constitute the chemical structure of the raw materials. This was confirmed by EDS analysis, which is included in Figure 3 and presented in Figures S2–S4, respectively. Given that CaO was utilized during the synthesis of PwP and PwPL, the EDS figures also demonstrate the presence of calcium.

### 3.2. In Vitro Measurement of the Bioaccessibility Rate between Pectin and Total Carotenes

The DC levels in the supernatants of the duodenal chyme were 223.8 mg g^−1^ and DCP was 71.1 mg g^−1^. Therefore, the bioaccessibility was 31.7%.

### 3.3. Rate of Pectin Secretion Evaluated at Treatments and pH Differences

The secretion of D-galacturonic acid (uronic acids, µg) between pH levels (rows) showed differences between treatments when analyzed with pH levels. The PnP had the highest values with a mean of 97.34. PwP then had 16.24, and PwPL had 20.32. The differences between each treatment (columns) indicate that the protected pectin quantified by D-galacturonic acid was greater at pH 2 and 3 vs. pH 5 and 7 (*p* < 0.05); the release increasing by 65 and 52% between pH = 3 vs. pH = 7 in the PwP and PwPL treatments (*p* < 0.05), respectively (Table 1).

### 3.4. In Situ Degradability of Pectin with Two Protection Techniques

The in situ degradability percentage of the citrus pectin protected with the two evaluated techniques showed differences (*p* < 0.05). The unprotected citrus pectin (PnP) degraded by 99% at 24 and 48 h, while the PwP was 27.59% at 24 h and 29.23% at 48 h. Protection by immersion with shellac and PwPL had less degradability at 24 and 48 h of incubation of 16.75% and 18.60%, respectively (Table 2).

### 3.5. Effect of the Use of Protected Pectin on the Chemical Profile of Milk from Grazing Cows

The use of protected citrus pectin in feeding dairy cows increased (*p* < 0.05) the percentage of fat in milk (4.588 vs. 3.295%). Total solids increased by 17% using protected pectin (8.2 vs. 6.96%). The treatments did not differ in protein content, density, brix degrees, or pH (Table 3). Protected citrus pectin in cow feed had a similar L* value in the two treatments. The value a* (NP = −5.5418, PwPL = −2.9015) and value b* (NP = 7.5685, PwPL = 2.8063) had significant differences (*p* < 0.05), while the c* value had no significant difference (*p* > 0.05) (Table 4). Protected citrus pectin in dairy cows decreased (*p* < 0.05) the blood concentration of glucose (NP = 56.80 vs. PwPL = 42.60 mg dL^−1^), cholesterol (NP = 167.10 vs. PwPL = 151.10 mg dL^−1^), and lactate (NP = 3.13 vs. PwPL = 1.46 mg dL^−1^). There were no differences in blood triglyceride levels (Table 5).

## 4. Discussion

Fabricating microparticles has emerged as an important research field to resolve challenges in agriculture, industry, electronics, and biomedicine. However, the advantages they represent depend upon several parameters such as size, shape, surface charge, and chemical composition.

UV-Vis analyses of microparticles are required to evaluate the agglomeration state, presence of functional groups, and approximate size and shape of microparticles. Here, we observed that PnP microparticles exhibited a higher absorption peak, which can be attributed to the uncovered presence of pectin. However, this band decreased once palm oil and shellac were incorporated. FTIR spectroscopy was employed to confirm the adequate integration of pectin, palm oil, and shellac within the developed microparticles. As depicted in the results section, PnP, PwP, and PwPL microparticles exhibited similar peaks within the range of 3500 and 700 cm^−1^ that correspond to the functional groups in the chemical structure of the raw materials. Some of these groups included hydroxyl, methyl, and carbonyl groups.

Using DLS, the average size, ζ-potential, and PDI of PnP, PwP, and PwPL were recorded. DLS, also known as photon correlation spectroscopy, was implemented to determine the average hydrodynamic size, size distribution, ζ-potential, and polydispersity index (PDI) of objects that can range from micrometers to nanometers. In biological systems, several studies have demonstrated that small nanostructured materials (<100 nm) can exhibit enhanced cellular uptake, interaction with biomolecules, biological performance, circulation time, and biodistribution [37]. Comparably, it has been documented that the size of microparticles can influence their capacity to release and deliver entrapped substances [38], distribution, and cellular uptake [39]. The scientific evidence regarding using microparticles or nanoparticles to mitigate rumen degradation is scarce. However, recent reports have demonstrated that microspheres fabricated with citrus pectin and calcium pectinate containing urea downregulated the risk of ruminant intoxication without affecting the ruminal pH and temperature [40]. Comparatively, starch and gum Arabic-maltodextrin microparticles containing *Acacia mearnsii* extract have been demonstrated to decrease methane emissions in vitro [41].

The ζ-potential refers to the degree of electrostatic repulsion between adjacent particles with similar charge. It is a crucial parameter to evaluate microparticles and nanoparticles as they indicate their stability in solution and utility in the future design of micro- and nanotechnologies [42]. It was recently documented that microparticles with a ζ-potential higher than +30 mV are prone to be stable in suspension as they prevent aggregation [43]. However, this can also apply to negatively charged microstructures, which is the case of PnP (−50.2 mV), PwP (−22.5 mV), and PwPL (−11.5 mV) microparticles. The negative charge of the developed microparticles can be attributed to the fact that dissolved pectin at neutral pH is negatively charged.

The PDI of microparticles is used to determine their uniformity in solution. In this sense, higher PDI values (>0.7) indicate the tendency of microparticles to aggregate and then form a multiple particle size population that can be unstable and unsuitable for further applications. On the contrary, lower PDI values (<0.05) are representative of monodisperse, homogenous, and stable microparticles [38]. The results through DLS evaluation demonstrated that PnP, PwP, and PwPL microparticles are monodisperse and stable structures during analysis as they exhibited PDI values < 0.05. The PDI values of PnP, PwP, and PwPL microparticles can be consulted in the supplementary material of this study. The morphology of these samples was observed with SEM.

Among electron microscopy approaches, SEM is necessary to observe microparticles’ morphology, dispersion, and structure. During the analysis of samples, it was noted that PnP, PwP, and PwPL microparticles possess inconsistent shapes as they were ovoid or completely amorphous. In addition, it was observed that they are wrinkled, hollow, and slightly spherical, which are features that can enhance their adhesion to surfaces or permeability within biological components. These results are challenging to compare to the recent scientific literature as the structural arrangement of microparticles relies on the synthesis method, experimental conditions, and raw materials used for their preparation.

Various endogenous and exogenous characteristics influence the slow release of particles made with polymers. These characteristics include particle size, morphology, porosity, hygroscopicity, hydrophobicity, surface tension, and thermal action [39]. Shellac coatings strengthen the substrate and reduce absorption. Limiting the absorption is essential for the protection capacity and uniformity to improve the high performance of the microparticles. The shellac coating resists water absorption and prevents its swelling due to humidity. In addition, some larger particles had a hexagonal shape, similar to what has already been observed for other nanometer-sized zirconia-based materials [29,39].

However, when designing particles specifically for degradation in the digestive tract of ruminants, it is essential to focus on factors that facilitate the release of drugs or additives. In this case, pH sensitivity is the primary variable that influences the particle degradation [40], and it can be controlled through polymer coating. In our study, pectin particles protected with palm oil and shellac (PwPL) demonstrated satisfactory performance, degrading within the pH range of 2 to 5 with greater efficiency at pH 3; this response is due to the concentration of hydrogen ions released and the gelation of pectin in the small intestine [16]. Similar studies have successfully protected other substances, such as *Lactobacillus plantarum* and omega-3 fatty acids, using alginate and pectin [41] or employing shellac as a coating material in the production of urea-formaldehyde resin microcapsules [42]. These studies achieved effective protection. However, the morphology and size of the particles depend on the encapsulation technique and the materials used for coating the particles [39,43]. In our study, the particles protected with shellac exhibited greater firmness, but their size was larger due to the volume increase caused by shellac, reaching up to 439 μm [44]. The hardness and mechanical resistance of the particles influence their degradability. Therefore, it is necessary to incorporate the polymer gradually to allow for the desired degradation effect of the particle in response to pH.

Pectin antagonizes β-carotenes; therefore, the particles must not degrade in the rumen’s acidic pH. Instead, they should pass through the abomasum to be solubilized in the intestine. Theoretically, shellac is soluble in alkaline solutions but resistant to water. Its moldable nature under pressure, temperature, and dissolution in volatile organic solvents has led to its use in coatings known for their hardness and durability [40,45]. In this study, the results indicate that the maximum release of protected citrus pectin occurs at pH 3.0, supported by the resinous properties of shellac. These findings suggest that particles protected with the polymer can effectively encapsulate lipophilic compounds in the intestine [46].

Previous research has reported encapsulation of Lactobacillus reuteri [47] and Lactobacillus paracasei [48], utilizing whey powder, sodium alginate, and shellac coatings, resulting in a 77% improvement in probiotic survival at pH 2. Slow-release theophylline microcapsules coated with shellac have also been developed [45], and their evaluation at pH 1.2 revealed minimal gastric degradation within the first 120 min.

In ruminants, spray-dried microcapsules composed of porous starch as the core material, triple-coated with Eudragit E100 and Shellac, have been prepared. These microcapsules had a particle diameter of 20–30 μm and demonstrated stability in neutral rumen pH (pH 6.5). The degradation efficiency of these microcapsules reached 85% within 30 min at pH 3.0 [32]. As a polymer, Shellac increases porosity and is associated with particle size [48]. In our study, the pectin’s morphology allowed for the incorporation of shellac into the matrix, resulting in increased particle porosity and enhanced release of D-galacturonic acid at acidic pH. The pectin’s resistance against proteases, amylases, and ruminal microorganisms made the particle dosage appropriate [49]. Shellac has been used to protect against moisture in the controlled administration of drugs and nutrients due to its ability to form a protective film. Additionally, the polymer is non-toxic and biodegradable [50].

The color of milk is influenced by several extrinsic and intrinsic factors, with diet, the amount of fat globules, and pigment storage having a significant impact [51]. The use of protected pectin particles improved the milk fat content, thus increasing the total solids content. Although no studies indicate a change in fat metabolism in the mammary gland due to pectin, the encapsulation/protection process of palm oil, used as a protectant, influenced the increase in milk fat content. Published studies [52,53] suggest that fatty ingredients in cow feed can increase low-density lipoprotein content in blood plasma directed towards the mammary gland and reduce the synthesis of short-chain fatty acids, consequently increasing the total fat in milk. Palm oil has been utilized as a protective agent against rumen microorganism degradation of particles and to enhance milk fat content. Some studies have shown that supplementing with 200 g of protected palm oil increased milk production [54] and total solids content [55,56].

Milk color is an effective traceability method, allowing the determination of the cow’s feeding system, and is associated with milk quality [11]. In our study, the protected pectin particles decreased the b* value, related to the degree of yellow coloration in milk. The yellowish color in grazing cows is mainly attributed to the high carotenes present in pastures [57]. Our results indicate that pectin is an effective antagonist for β-carotenes, preventing their absorption, accumulation in the mammary gland, and subsequent excretion in milk [18]. Bovine cattle commonly exhibit a more yellowish milk color compared to goat milk. Goat milk contains less vitamin A (1560 IU/L vs. 2074 IU/L). Goats metabolize carotene into vitamin A at the intestinal level, resulting in the absence of carotenes in goat milk [58]. Physiologically, cattle lack the enzyme 15, 15’-monooxygenase at the intestinal level, which hinders the conversion of β-carotene isomers into vitamin A [10]. The breakdown of β-carotene generates two molecules of retinaldehyde, which are then converted to retinol and further to vitamin A [59].

The cholesterol, glucose, and lactate levels found in the cows in this study align with previous research conducted by our research group [18]. The study indicates that hematic cholesterol levels in calves decreased by 15, 18, and 27% as the pectin dose increased from 18.5 to 46.25 and 92.5 g/day. The pectin supplement induces hypocholesterinemia, possibly by blocking cholesterol absorption or reducing enterohepatic circulation. Both mechanisms interfere with bile acid absorption, as observed in studies conducted on humans [60], chickens [13], and rats [61]. These studies suggest that the low levels of pectin in plasma and the liver result from inhibiting bile acid absorption decreasing cholesterol absorption. Pectin significantly inhibits blood cholesterol levels by interrupting micelle formation, reducing bile acid diffusion, and blocking cholesterol-carrying micelle absorption [62]. In mice with normal or elevated lipid levels, a daily consumption of 6 g of pectin reduces cholesterol levels, thus reducing the risk of coronary heart disease [63].

Studies by Saikia and Gogoi [64] have demonstrated that pectin derived from prickly pears affects hepatic cholesterol homeostasis in guinea pigs. Pectin also affects blood glucose levels and possesses potential anti-diabetic properties, as observed in studies conducted on diabetic rats, where it proved effective treatment of type 2 diabetes mellitus [65,66]. Citrus pectin improved hyperlipidemia, liver glycogen content, and glucose tolerance in diabetic rats [65]. Research by Wathoni et al. [67] demonstrated that methoxylated apple pectin can be a functional ingredient to reduce insulin resistance. Another study revealed that pectin supplementation in rats with metabolic syndrome improved glucose homeostasis, with the hypoglycemic effect attributed to its ability to absorb water and form gels in the small intestine, trapping sugars and reducing their absorption [68].

In the small intestine, pectin forms viscous gels, as observed in our study. Pectin can retain water due to its viscosity, and it also reduces the reabsorption of bile salts, preventing cholesterol formation and the action of lipases [69]. Our previous research has shown that pectin infusion in the duodenum reduces blood cholesterol levels and total fat excretion in bovine feces. The effect is attributed to lipids’ physical entrapment by forming a high methoxyl pectin gel, which promotes hydrophobic interactions between pectin and lipids.

## 5. Conclusions

The process of forming pectin microparticles coated with shellac was successful. The SEM results showed that the particles vary their morphology according to the combination of encapsulating and coating materials. SEM analyses showed that the pectin had been protected and an amorphous state formed with the shellac microparticles. The analysis of PwPL microparticles indicated that pectin was completely covered by shellac and palm oil and exhibited a sharp peak at 3641 cm^−1^ with a wide band at 3326 cm^−1^, which corresponded to the stretching vibration of the O–H bond from pectin. In addition, designing particles protected with lipids and polymers is an economical method that resists degradation at pH levels greater than five. The designed particles exhibited irregular, rough, and porous shapes, with the morphology and texture dependent on the polymer used. These protected particles were effectively utilized in ruminants since it was found that protected pectin was greater at pH 2–3 with an increase in secretion of ~58%. For instance, the inclusion of 100 g of pectin per animal per day in the feed of grazing dairy cows resulted in a reduction in yellow coloration (b* = 2.8) of milk, and the physicochemical characteristics of the milk grains remained unaffected.

## Figures and Tables

**Table 1:** Release of pectin as D-galacturonic acid (uronic acids, µg) at treatments and pH differences.

pH	PnP	PwP	PwPL	SE
2	99.685 ^a,x^	18.961 ^c,xy^	21.979 ^b,xy^	1.013
3	99.535 ^a,x^	23.380 ^c,x^	27.581 ^b,x^	1.287
5	97.337 ^a,x^	14.451 ^c,y^	18.408 ^b,y^	1.567
7	92.816 ^a,y^	8.163 ^c,z^	13.311 ^b,z^	1.614
SE	0.99	1.131	1.121	

^a–c^ Different superscripts between files indicate significantly different (*p*  < 0.05). ^x–z^ Different superscripts between columns indicate significantly different (*p*  < 0.05). Abbreviations: SE: standard error of mean; PnP: unprotected citrus pectin; PwP: citrus pectin protected with palm oil; and PwPL: citrus pectin protected with palm oil and shellac.

**Table 2:** In situ degradability percentages in protected pectin particles.

Treatment	24 h	48 h
PnP	99.42 ^a^	99.23 ^a^
PwP	27.59 ^b^	29.23 ^b^
PwPL	16.75 ^b^	18.60 ^b^
SE	6.24	6.08
*p* value	0.0001	0.0001

Abbreviations: SE, standard error of mean; PnP, unprotected citrus pectin; PwP, citrus pectin protected with palm oil; PwPL, citrus pectin protected with palm oil and shellac; ^a,b^, means with different literal between columns are statistically different (*p* < 0.001).

**Table 3:** Effect of protected pectin on the chemical profile of milk in grazing cows; the levels of fat, total solids, protein, and lactose are expressed in %, whereas the density values are expressed in g/mL.

Treatments	Fat	Total Solids	Density	Protein	Lactose	Degrees Brix	pH
NP	3.295 ^b^	6.957 ^b^	28.150	3.120	4.417	9.095	7.200
PwPL	4.588 ^a^	8.200 ^a^	31.002	3.210	4.860	8.647	7.297
SE	0.379	0.409	1.098	0.104	0.166	0.295	0.034
*p* value	0.001	0.0033	0.0516	0.391	0.094	0.1337	0.064

Abbreviations: SE, standard error of mean; NP, no citrus pectin in the supplement; PwPL, citrus pectin protected with palm oil and shellac; ^a,b^, means with different literals in rows are statistically different (*p* < 0.01).

**Table 4:** Effect of the use of protected pectin on the color of milk in grazing cows.

Treatments	L	A	B	C
NP	80.5169	−5.5418 ^a^	7.5685 ^a^	81.0884
PwPL	80.1193	−2.9015 ^b^	2.8063 ^b^	80.2273
SE	0.9972	0.1986	0.3275	0.9995
*p* value	0.6918	0.0001	0.0001	0.3916

Abbreviations: SE, standard error of mean; NP, no citrus pectin in the supplement; PwPL, citrus pectin protected with palm oil and shellac; chromatic area L* = brightness (0 is black and 100 is white); chromatic area a*: ranges of redness, (<) green, and (>) red; chromatic area b*: range of yellowness, (>) corresponds to yellow and (<) corresponds to blue; C*, chroma is defined as (a*)2 + (b*)2; ^a,b^, means with different literals in columns are statistically different (*p* < 0.001).

**Table 5:** Effect of the use of protected pectin on blood metabolites; glucose and cholesterol values are expressed in mg/dL whereas lactate and triglycerides are expressed in mmol/L.

Treatments	Glucose	Cholesterol	Lactate	Triglycerides
NP	56.80 ^a^	167.10 ^a^	3.13 ^a^	106.50
PwPL	42.60 ^b^	151.10 ^b^	1.46 ^b^	103.90
SE	4.68	1.06	0.24	1.57
*p* value	0.0071	0.0001	0.0001	0.1162

Abbreviations: SE, standard error of mean; NP, no citrus pectin in the supplement. PwPL: citrus pectin protected with palm oil and shellac; ^a,b^, means with different literals in columns are statistically different (*p* < 0.01).

## Data Availability

Data are contained within the article.

## Supplementary Materials

The following supporting information can be downloaded at https://www.mdpi.com/article/10.3390/polym16010106/s1, Figure S1: Manufacturing process of PnP, PwP, and PwPL microparticles; Figure S2: EDS analysis of PnP microparticles; Figure S3: EDS analysis of PwP microparticles: Figure S4: EDS analysis of PwPL microparticles.
