# Supplementary material for: Enhancing Pectin Particles with Polymer Additives: Mitigating Rumen Degradation and Minimizing Yellowish Milk Color in Grazed Cows
Source: Polymers (Basel). 2023 Dec 29;16(1):106. doi: 10.3390/polym16010106 (PMC10780586; doi:10.3390/polym16010106)
Supplement: Supplementary file 1 [file polymers-16-00106-s001.zip › polymers-2749180-supplementary.pdf]

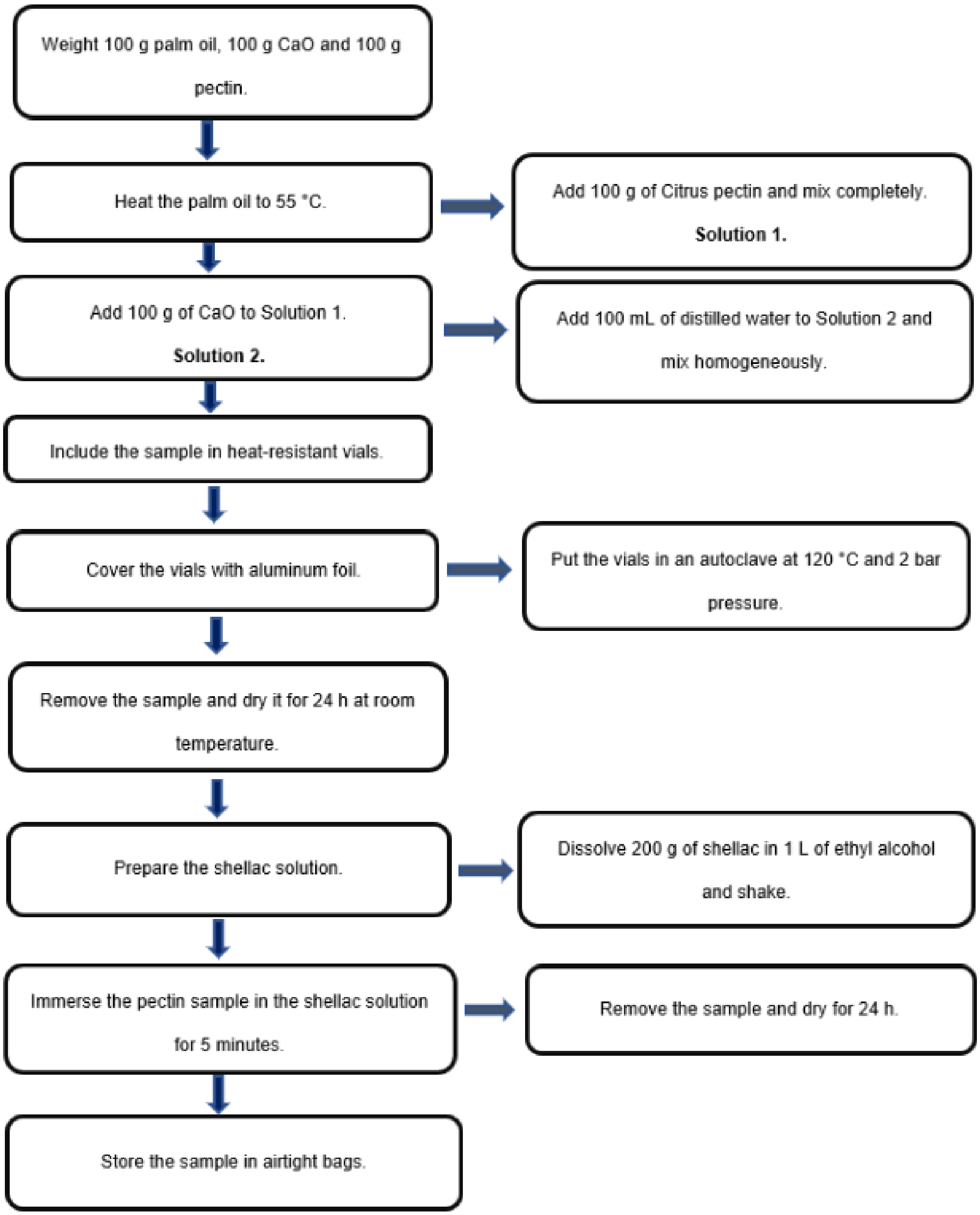

Figure S1. Manufacturing process of PnP, PwP, and PwPL microparticles.

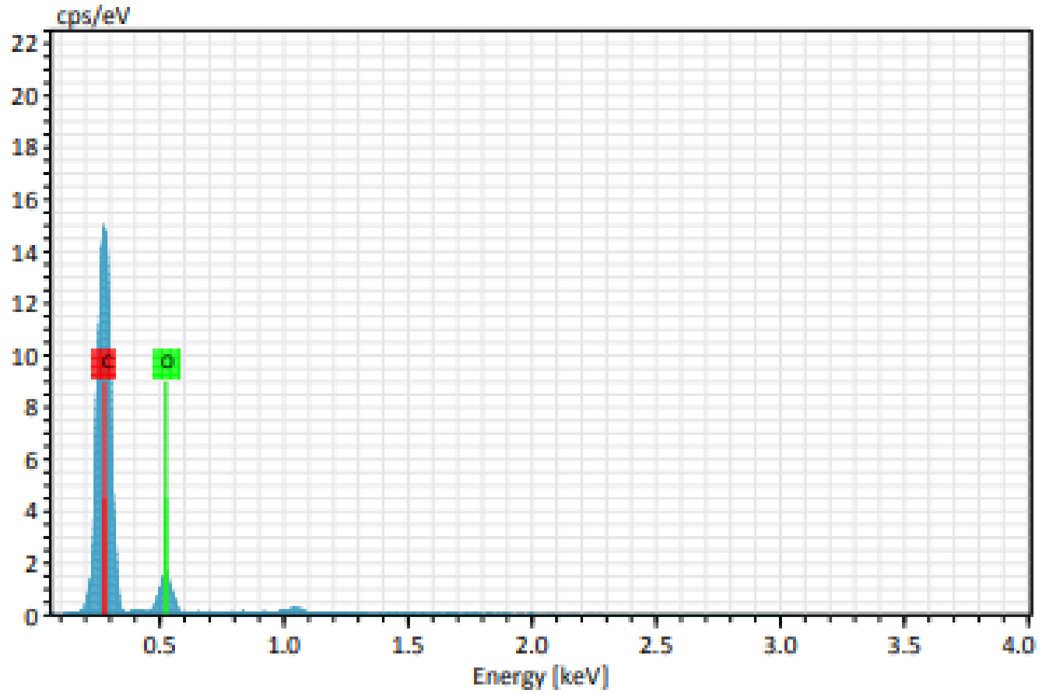

Figure S2. EDS analysis of PnP microparticles.

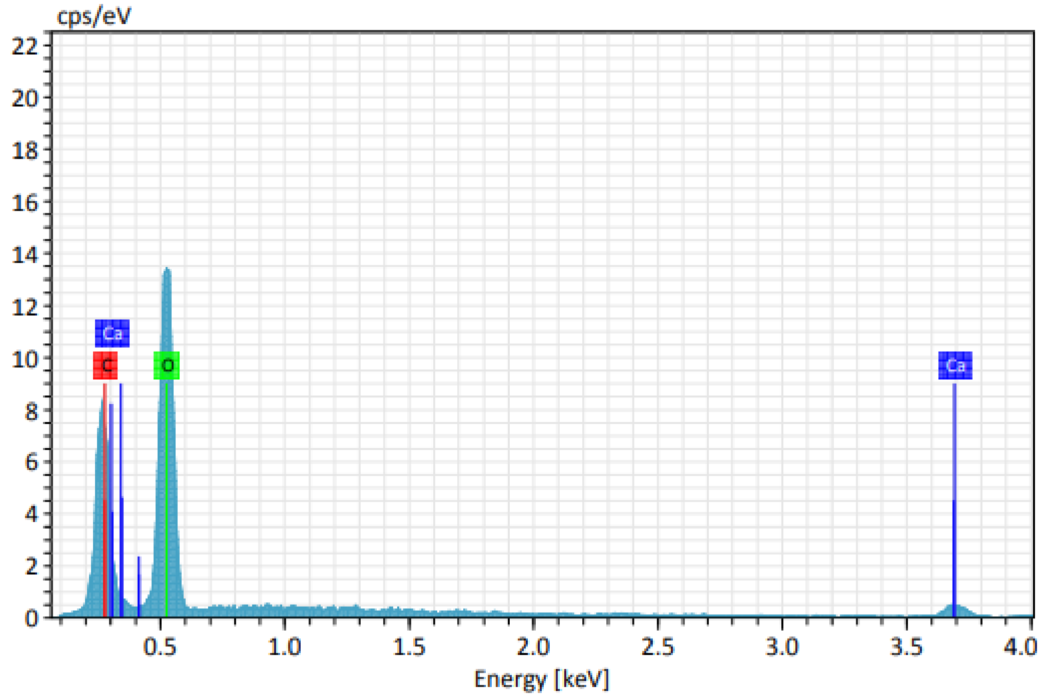

Figure S3. EDS analysis of PwP microparticles.

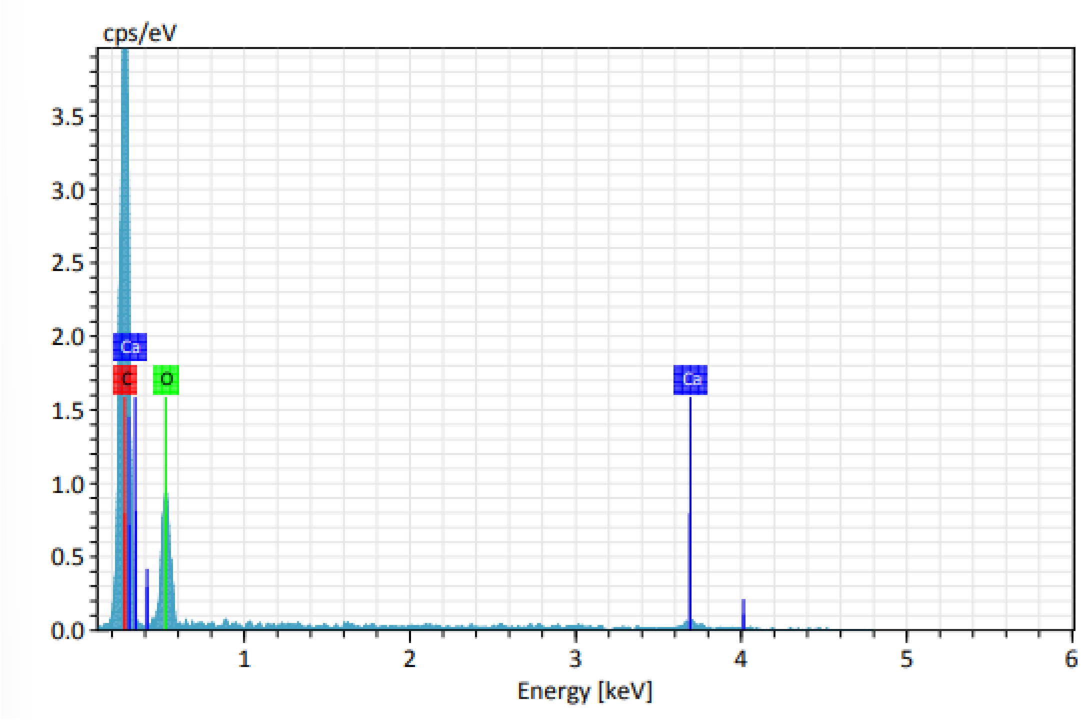

Figure S4. EDS analysis of PwPL microparticles.
